# Early Detection and Remote Care of Frailty for Older Adults Living in Sparsely Populated Rural Areas in Hokkaido, Japan: A Case Study

**DOI:** 10.7759/cureus.84194

**Published:** 2025-05-15

**Authors:** Kazuki Yokoyama, Shuhei Fukagawa, Hideyuki Tashiro, Hajime Toda, Takuto Kida, Lin Takahashi, Atsushi Mizumoto, Keitaro Makino, Kaori Yokoyama, Tomomi Akanuma, Satoshi Kondo, Hikaru Ihira

**Affiliations:** 1 Department of Occupational Therapy, School of Health Sciences, Sapporo Medical University, Sapporo, JPN; 2 Department of Nursing, School of Health Sciences, Sapporo Medical University, Sapporo, JPN; 3 Department of Physical Therapy, School of Health Sciences, Sapporo Medical University, Sapporo, JPN; 4 Division of Cohort Research, Institute for Cancer Control, National Cancer Center, Tokyo, JPN; 5 Graduate School of Health Sciences, Sapporo Medical University, Sapporo, JPN; 6 Major of Physical Therapy, Department of Rehabilitation, Faculty of Healthcare and Science, Hokkaido Bunkyo University, Eniwa, JPN; 7 Center for Environmental and Health Sciences, Hokkaido University, Sapporo, JPN; 8 Department of Health and Welfare Aged Care Division, Bibai City Office, Bibai, JPN; 9 Graduate School of Engineering, Muroran Institute of Technology, Muroran, JPN

**Keywords:** activities of daily living, artificial intelligence-assisted monitoring system, frailty, loneliness, multidisciplinary care, older adults, psychological well-being, remote care, social emotional support, sparsely populated rural areas

## Abstract

The global increase in the number of older people aged 65 and over is causing concern in healthcare and social systems. The lack of health and welfare services and human resources may delay the assessment of frailty and other geriatric syndromes. However, the participation in community-led frailty check-ups remains low, and there are limitations in providing a preventive home visit approach to all residents. Therefore, efficient remote frailty assessment and care systems to support older people in sparsely populated rural areas are needed. This case study investigated the efficacy of an artificial intelligence (AI)-assisted monitoring system coupled with remote multidisciplinary care in addressing frailty among older adults in a depopulated region of Hokkaido, Japan. An 85-year-old woman, residing in a sparsely populated rural city with a significantly high aging rate, was assessed for frailty using the revised Japanese version of the Cardiovascular Health Study criteria. In addition, three aspects of frailty were assessed: physical frailty, cognitive/psychological frailty, and social frailty. Baseline assessments indicated prefrailty, diminished mobility, depressive symptoms, low subjective well-being, and social isolation. An AI-assisted monitoring camera was installed in the participant’s living space to provide continuous behavioral analysis. Based on the abovementioned information, multidisciplinary remote care was provided. Over two months, the system identified mobility challenges and prolonged sedentary behaviors, despite no falls or emergencies. In remote care, multidisciplinary teams suggest exercises and environmental adjustments to improve physical activity and activities of daily living, as well as social participation to maintain a sense of purpose and roles in life. Through these interventions, three months after baseline, while her physical frailty progressed, her psychological well-being and social emotional support showed improvements. Notably, she expressed a sense of security with the presence of the monitoring system and appreciated the remote care advice, highlighting its role in alleviating feelings of isolation. This case demonstrates the potential of integrating an AI-assisted monitoring system with remote care to mitigate the multi-dimensional effects of frailty in aging populations, particularly in regions facing disparities in healthcare access. Our findings suggest that such systems can provide valuable insights into daily behaviors, facilitate tailored interventions, and foster a sense of safety among older adults living in sparsely populated rural areas.

## Introduction

The escalating global population of older adults aged 65 and older poses considerable challenges to healthcare and social systems, as well as inequalities in health [1]. Population aging is a global challenge. Japan recorded the highest proportion of older adults in the world [2]. The regional disparity in Japan’s aging population is a pressing issue. Urban areas, such as Tokyo, Osaka, and Sapporo, have relatively better access to healthcare and welfare services than rural regions that face challenges such as depopulation and limited resources. Approximately 29.3% of the inhabitants are 65 years and older, and 17.0% are 75 years and older in 2024 [3], with sparsely populated rural areas having a higher proportion of older adults than urban centers [2]. This disparity is exacerbated by factors such as insufficient transportation options and a lack of medical facilities in sparsely populated rural areas, making it difficult for older adults to access essential services [2]. Furthermore, the Ministry of Health, Labour and Welfare highlights that healthcare and caregiving resources are unevenly distributed across regions. For instance, the availability of nursing homes and specialized medical care is significantly lower in sparsely populated prefectures [4]. In addition, even with the use of preventive interventions, services are expected to be less accessible in sparsely populated rural areas.

Frailty is an age-related clinical syndrome whose prevalence rises with age, characterized by increased risk of adverse health effects, including death, institutionalization, falls, and hospitalization, with pre-frailty defined as the preliminary stage [5]. It has been indicated that frailty is reversible to a donative state with appropriate intervention and good lifestyle habits [6,7]; therefore, it is important to identify frail older people at an early stage and to take action as soon as possible. However, a meta-analysis of older adults living in rural communities found that the prevalence rates of frailty and pre-frailty were 18% and 50%, respectively, which are higher when the analyses are not confined to rural communities [8]. Addressing these disparities requires tailored policies such as improving transportation infrastructure in rural depopulated areas and promoting community-based support systems.

In Japan, frailty health checkups, particularly for individuals aged 75 and older, have been implemented as part of efforts to prevent and manage frailty. However, the participation rate remained relatively low: per one report, only 23.1% of elderly individuals eligible for frailty health checkups underwent the checkups, while 66.8% did not [9]. Thus, to provide healthcare to all residents, measures are required to deal with those who do not attend checkups. Although preventive home visit approaches have been considered, a systematic review showed that such support was not effective [10], and human resources in the community to provide such support are lacking [11]. Furthermore, in sparsely populated rural areas, supportive relationships with the surrounding community have decreased, and poor social support leads to increased hesitation in relying on others [12]. To address these problems, an efficient system is needed to automatically assess the frailty of older adults living in sparsely populated rural areas and provide remote care based on the results of the assessment. The detection of frailty using monitoring systems with AI sensors that recognize postural instability and falls, which have been associated with frailty [13,14], and subsequent remote notification of the event may lead to the implementation of appropriate remote care. In our case study, an AI-assisted monitoring system was used to assess frailty in an older adult living in a sparsely populated rural area of Hokkaido, Japan. Additionally, remote care was provided by a multidisciplinary team based on the assessment results.

## Case presentation

Guideline

This report was prepared in accordance with the CARE (CAse REport) guideline [15].

Participant information

The participant was an 85-year-old Japanese woman residing in Bibai City, Hokkaido, Japan. Her height, weight, and body mass index were 154.1 cm, 72.3 kg, and 30.4, respectively. She lived with four family members but spent time alone during the day. She was certified under Japan’s national long-term care insurance and was assessed as care level 1, which is the level of need for partial care in daily life; it is the shortest of the five levels of care required, taking between 25 and 32 minutes a day [16,17]. She goes to a day service center with long-term care insurance twice a week. However, she spent most of her time at home, especially in her room. Regarding the household, she was responsible for laundry and meal preparation.

Bibai City, located in Hokkaido, Japan, has a population of approximately 20,000, decreasing 21.7% over the past 10 years. It lies approximately 60 km from Sapporo, making it easily accessible within an hour by car. Known for its coal mining history and agricultural landscapes, Bibai City faces significant demographic challenges, with an aging rate of 44.3% in 2024, far exceeding the national average of 29.3% [18].

Written informed consent was obtained from a participant for this study and for the publication of this case report. This study was approved by the Sapporo Medical University Ethical Review Board (approval number: 5-1-49; approval date: November 9, 2023).

Procedures

**Figure 1:**
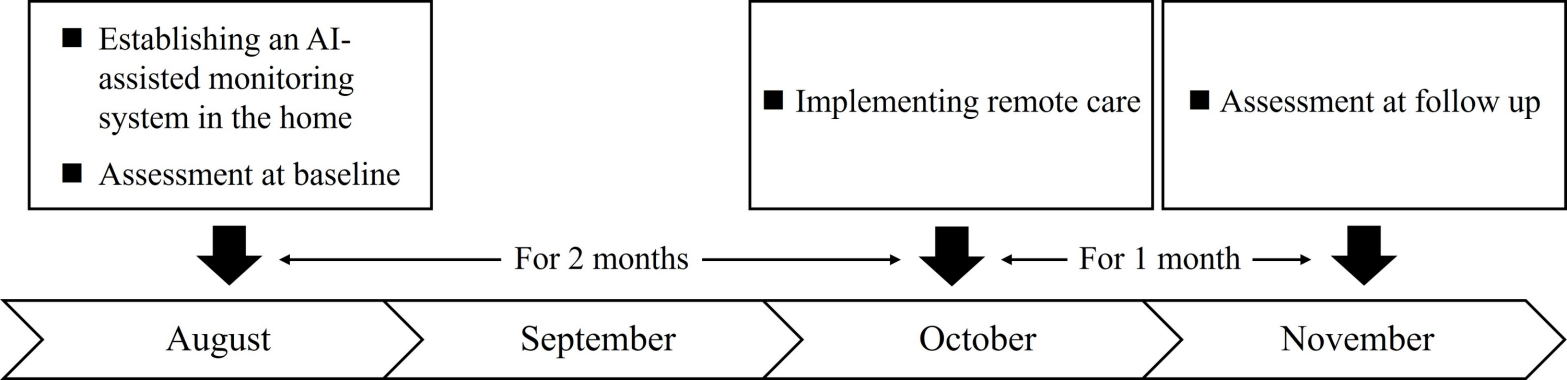
The survey flowchart

**Figure 2:**
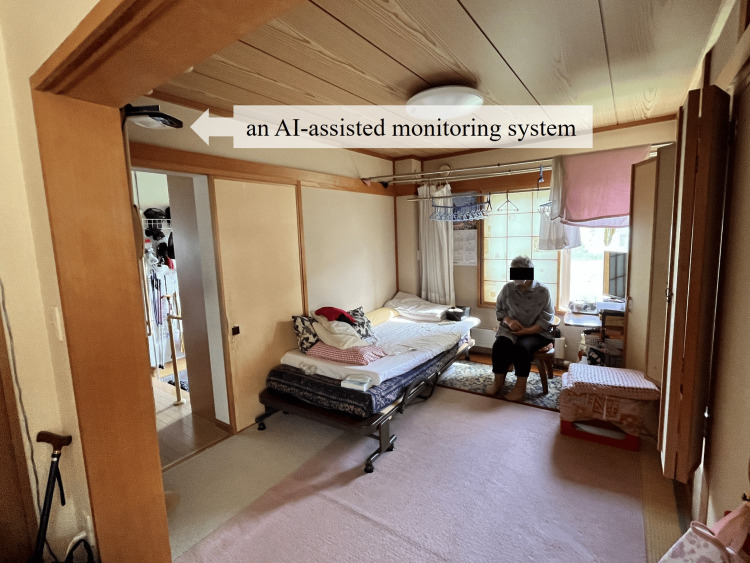
Installed an AI-assisted monitoring system in the living room and bedroom of her home

The survey flow is illustrated in Figure 1. At baseline, frailty, including three aspects: physical, cognitive/psychological, and social, and activities of daily living (ADLs), including basic and instrumental ADL, were assessed using tests, scales, and questionnaires in August 2024. Simultaneously, an AI-assisted monitoring system, HitomeQ Care Support (Konica Minolta, Inc., Tokyo, Japan) [19], was fitted to the ceiling of the room used as the living room and bedroom (Figure 2). This system uses AI image analysis to detect postural instability and falls recorded by a camera and analyze behavior. Specifically, it recognizes actions by estimating posture from joint points and assesses motor functions by identifying the speed and repetition of movements. It was used to identify frailty clues from daily life at home.

After two months of video monitoring, remote care was provided by a multidisciplinary team of physical therapists, an occupational therapist, a certified public psychologist, and a public health nurse based on the assessment of frailty at baseline and the results of a monitoring system. Changes in her behavior due to remote care were also assisted using a monitoring system.

After a one-month post-assessment monitoring period, a follow-up survey was conducted to assess frailty and ADLs. The participant and her family were also asked about their opinions regarding the AI monitoring device and remote care.

Assessment

Frailty is assessed using the revised Japanese version of the Cardiovascular Health Study (J-CHS) criteria [20]. Usual gait speed was measured as the time required to walk at a normal pace along a 6-m walking path with a preliminary section of 2 m each at the beginning and end in a hospital rehabilitation room. Grip strength was measured using a Smedley dynamometer (Matsumiya Medical Industry, Tokyo, Japan). Two trials were performed with the dominant hand, and the larger value was recorded. As frailty consists of three aspects - physical, cognitive/psychological, and social - each was assessed in detail in the present study. For assessing physical frailty, the timed up and go test (TUG) [21] and 25-question Geriatric Locomotive Function Scale (GLFS) [22] were used; for assessing cognitive/psychological frailty, the Mini-Mental State Examination (MMSE) [23], National Center for Geriatrics and Gerontology Functional Assessment Tool (NCGG-FAT) [24,25], the Geriatric Depression Scale (GDS) [26] and WHO-Five Well-Being Index (WHO-5) were used [27]; and for assessing social frailty, the Social Support Exchange Scale (SSES) for community-dwelling elderly [28] and Japanese version of the abbreviated Lubben Social Network Scale (LSNS) were used [29]. Her ADLs, Barthel Index [30], and Frenchay Activities Index [31] were also determined. Furthermore, the participant’s movements and activities in daily life were assessed by AI-assisted automated judgments and video confirmation using a monitoring system.

Results of the assessment at baseline

The results of the assessment at baseline are presented in Table 1 for frailty, according to the revised J-CHS criteria, and in Table 2 for physical, cognitive/psychological, and social functions (see results at baseline for each). The participant was judged to be pre-frail at baseline, with weight loss observed according to the J-CHS criteria. In the physical frailty survey, neither grip strength, gait speed, nor the TUG test met the criteria for being at risk. However, the GLFS-25 showed progressive decline in mobility [22], with difficulties in dressing and undressing, ascending and descending stairs, and walking without rest. Her MMSE score was 29 points, which was deemed normal [23]. The detailed cognitive function test (NCGG-FAT) showed higher scores than the reference scores for her age group [24,25]. The GDS [26] and WHO-5 index [27] showed a tendency toward depression and low subjective well-being. Regarding social frailty, her instrumental and emotional social support levels were lower than those reported in a previous study [28]. The LSNS revealed that her social networks included few connections with outside family members. Her basic ADLs were classified as independent functional status and instrumental ADLs, as partly independent functional status; she did not use transport, go out, perform heavy lifting, or do garden work. Her main complaints were that she experienced difficulty walking long distances, she needed to rest at regular intervals while moving around, and she experienced difficulty ascending and descending the stairs by herself.

**Table 1:** Assessment of frailty according to revised J-CHS criteria J-CHS: Japanese Version of the Cardiovascular Health Study Source: [20]

Component	Assessment	Baseline	After three months
Shrinking	Have you unintentionally lost 2 kg or more in the past six months?	Yes = 1	Yes = 1
Low activity	Do you engage in low levels of physical exercise aimed at health?	Yes = 0	Yes = 0
Exhaustion	In the past two weeks, have you felt tired without a reason?	No = 0	Yes = 1
Weakness	Grip strength < 18 kg in women	No = 0	No = 0
Slowness	Gait speed <1.0 m/s	No = 0	Yes = 1
Judgement	0 points: robust, 1-2 points: pre-frailty, 3-5 points: frailty	Pre-frailty	Frailty

**Table 2:** Assessment of physical, cognitive/psychological, and social functions ADL: Activities of Daily Living, BI: Barthel Index, FAI: Frenchay Activities Index, GDS: Geriatric Depression Scale, GLFS: 25-Question Geriatric Locomotive Function Scale, LSNS: Lubben Social Network Scale, MMSE: Mini-Mental State Examination, NCGG-FAT: National Center for Geriatrics and Gerontology Functional Assessment Tool, SSES: Social Support Exchange Scale, WHO-5: WHO-Five Well-Being Index

Category	Assessment	Baseline	After three months
Physical functions	Grip strength (kg)	25.5	26.5
	Gait speed (m/s)	1.09	0.94
	Timed up go test (s)	9.6	12.1
	GLFS (range 0-100)	67	71
Cognitive/psychological functions	MMSE total score (range 0-30)	29	30
	NCGG-FAT: Word list memory, immediate recognition	9	9
	NCGG-FAT: Word list memory, delayed recall	4	6
	NCGG-FAT: Trail making test part A (s)	22	19
	NCGG-FAT: Trail making test part B (s)	38	34
	NCGG-FAT: Symbol Digit Substitution Task	56	43
	GDS (range 0-15)	5	9
	WHO-5 (range 0-25)	10	13
Social functions	SSES, instrumental support (range 6-24)	15	16
	SSES, emotional support (range 6-24)	6	11
	LSNS: Family members (range 0-15)	11	11
	LSNS: Friends/acquaintances (range 0-15)	2	2
ADL	BI (range 0-100)	100	100
	FAI (range 0-45)	19	19

Results of the assessment using an AI-assisted monitoring system

**Figure 3:**
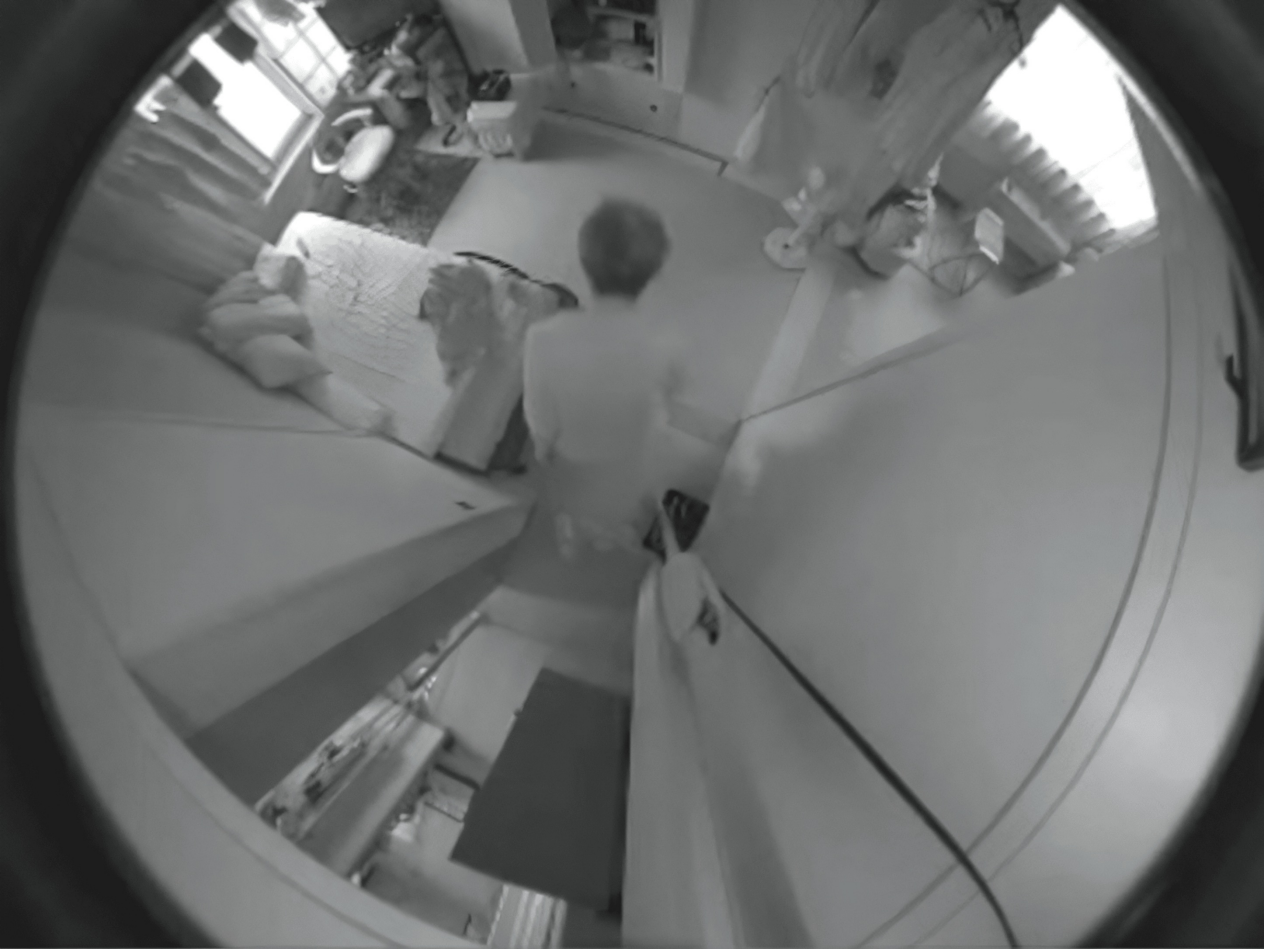
An example of an image captured using an AI-assisted monitoring system

**Figure 4:**
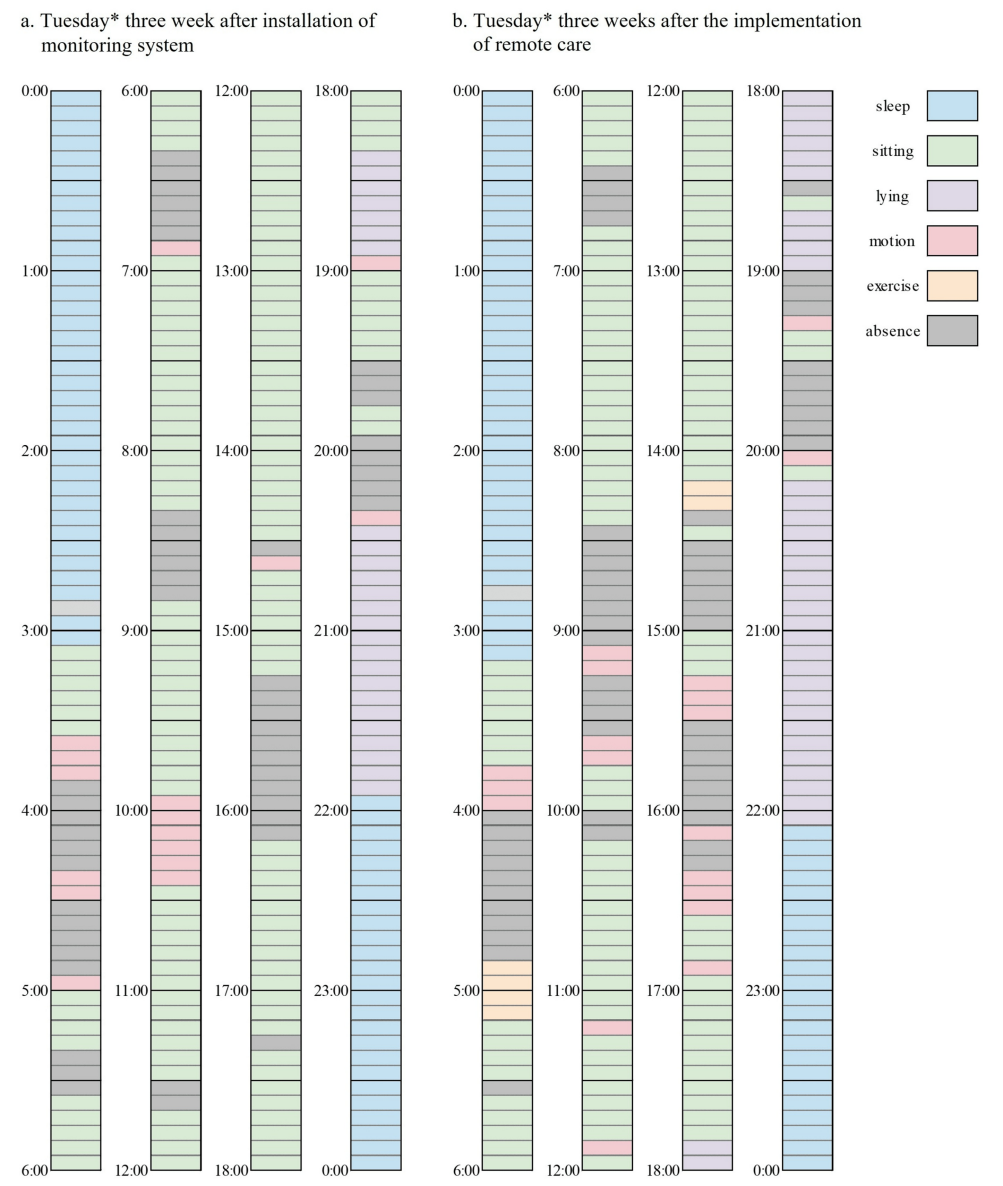
A 24-hour behavioral analysis using the monitoring system before and after remote care *Tuesday is a day when there is no day service, and the participant is alone during the day.

Data were collected for a total of three months: two months after the baseline survey and one month after the start of remote care. An example of an image captured using an AI-assisted monitoring system is shown in Figure 3. During this period, no falls or emergencies were reported, and the notification system was never triggered. However, the videos captured by the system indicated mobility problems. In one situation, on her way out of the toilet during the day, she tripped on a carpeted floor step at the entrance to the living room and almost fell forward. In another observation, she spent 90% or more of her waking hours lying on the bed or sitting in a chair in front of the table with a television on it. The result of the behavior analysis on Tuesdays without day service after three weeks of installation of a monitoring system is shown in Figure 4a. Furthermore, she took a long time to get up from the supine position and stand up from the sitting position; she would put on her trousers in the sitting position, and she had limited bending movements.

Remote care

**Figure 5:**
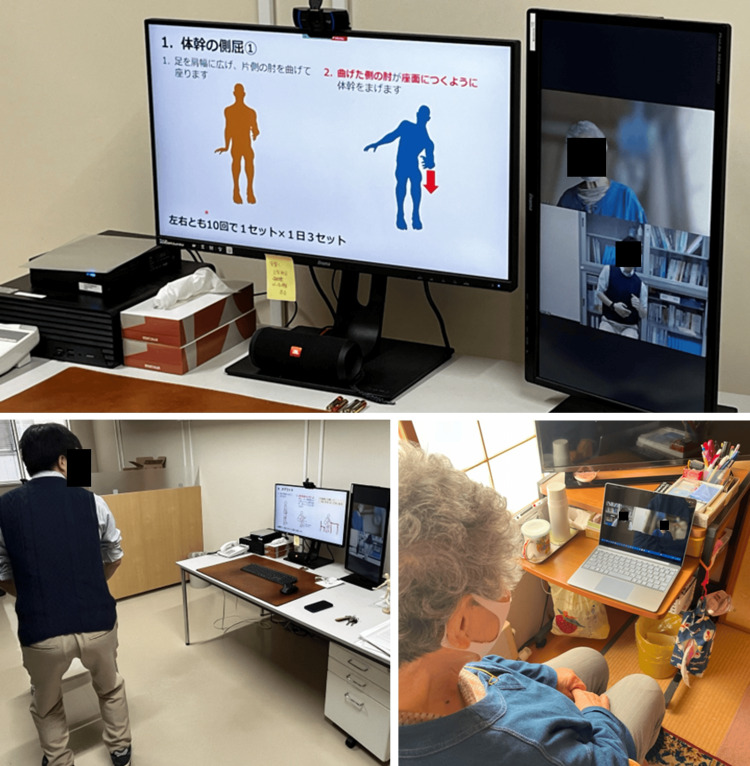
Implementation of remote care A presentation on trunk lateral flexion exercises is presented on the screen as part of the remote care. The procedure is shown as follows: “1. Sit with your feet shoulder-width apart and bend your elbow on one side,” and “2. Touch the bed with the right or left elbow and return to the starting position.” It is suggested that one set of 10 times on both sides should be carried out, three sets a day.

Two months after the baseline assessment, administrative staff, public health nurses, physical therapists, and occupational therapists implemented remote care based on the assessment of physical and mental functions, daily living evaluations, and investigations conducted via the monitoring system. Photographs of the implementation of online remote care are shown in Figure 5. Remote care was conducted for 40 minutes in a single session, connecting the multidisciplinary team via videophone and sharing slides showing exercise, environmental adjustments, and social participation to improve her physical function, depressive symptoms, and well-being, while interacting with the participants. Specific measures included locomotive syndrome prevention training to address reduced lower limb strength and balance, and core muscle training to combat decreased core strength. In addition, efforts were made to improve her depressive symptoms and well-being by proposing and exploring opportunities for outings, helping others, and increasing daily life activities.

Behavioral observations after remote care

The monitoring system showed that since the implementation of remote care, she engaged in 30 minutes of exercise, as suggested by the multidisciplinary team, every day, resulting in increased time spent exercising outside of day services. The results of the behavior analysis on Tuesday without day service after three weeks of remote care are shown in Figure 4b. The participant stated, "Through this monitoring system and remote care, if I'm connected to everyone, I can feel safe even when I'm alone," and "I used to never go shopping alone in winter, but this year, I was able to go out alone using trekking poles." Her family stated, "I think the awareness that there is monitoring is working, and she has gone from a sedentary life to being a bit more active."

Results of the post-remote care assessment

The results of the post-assessment at three months from baseline are presented in Table 1 for frailty according to the revised J-CHS criteria and in Table 2 for physical, cognitive/psychological, and social functions (see results after three months for each). In the follow-up survey, the J-CHS criteria for exhaustion and slowness were newly applied, and the participant was determined to be frail. The level of physical function also worsened in the TUG and GLFS, compared to the baseline. Cognitive function was maintained or improved compared to baseline, except for the symbol digit substitution task, which tested processing speed. Regarding psychological measures, depressive symptoms were worse than at baseline; however, well-being was higher than at baseline and previous study thresholds, showing a trend toward improvement. In terms of social frailty, the LSNS showed no change; however, the SSES indicated an improving trend in social support. ADL status showed no change over the three-month period. The frequency of service use and outings did not change. Her family stated, "Maybe feeling connected to other people through remote care has a positive effect on my loneliness."

## Discussion

This case study revealed the feasibility of installing an AI-assisted monitoring system in the homes of older residents in sparsely populated rural areas for assessing frailty in daily life and providing remote care. The distribution of local healthcare in Hokkaido is extremely uneven. This report is a first step toward addressing the need to assess frailty using AI-assisted monitoring systems and for early remote care by multidisciplinary teams.

In the case of our participant, both physical function and depressive symptoms showed a tendency to worsen over three months, leading to progressive frailty and negative outcomes, although there was no change in the ADL situation. However, appropriate interventions provided through remote care helped establish regular exercise habits. Behavioral changes were observed, such as exercising and making environmental adjustments, as suggested by remote care, even though the participant spent a lot of time in her own room. The participant and her family expressed positive views regarding the introduction of the AI-assisted monitoring system and remote care. Performing muscle strength or power exercises supervised by a trained professional can improve physical function; however, the effect is reduced when regular exercise is paused for some time [32]. Our participant was performing the exercises recommended during remote care; however, it is important to continue these exercises to improve physical function.

The participant’s scores on social support, particularly emotional support, and well-being improved throughout the study period. Perhaps the participant experienced an increased sense of social support by feeling connected to a multidisciplinary team that had a monitoring system in place and could provide appropriate care even when she was alone for long periods and despite having limited interpersonal relationships. Older adults with restricted social networks report low levels of emotional support [33]. Meanwhile, older adults with high emotional support report low levels of depression [34]; find ways to preserve and enrich family, friends, and community ties; and take advantage of neighborhood, community, and social activities [35]. The implementation of monitoring systems and remote care to create an emotional network, as in this study, may help improve mental health and contribute to increased motivation through preventive actions.

Research on remote care systems is still in its developmental stages. Falls and emergencies can be detected using AI, but determining frailty is difficult. In the future, studies can attempt to clarify which lifestyle behaviors detected by monitoring systems are associated with frailty to improve the accuracy of AI-assisted automated judgments. When using automated detection systems, it is important to check the consistency of the output of the system with the assessments made by experts, as done in this study, and to follow specific procedures to improve the validity of frailty assessments using an AI-assisted monitoring system. As a future perspective, early detection of frailty among older adults in sparsely populated rural areas who do not receive adequate support in the form of health checkups and visits can be promoted. To achieve this, AI-assisted monitoring systems that place as little psychological burden on older adults as possible are needed. The use of such strategies can help enhance frailty detection and remote care among residents who lack access to appropriate care.

## Conclusions

This case study demonstrated the feasibility of using an AI-assisted monitoring system and remote multidisciplinary care to assess and address frailty in older residents in a sparsely populated rural area. When physical function declined, the intervention improved social support and well-being, highlighting the potential of this approach to reach underserved populations. Future research should focus on enhancing AI-assisted frailty assessments and minimizing the psychological burden to expand the reach of remote care.
